# Why is women’s utilization of a publicly funded health insurance low?: a qualitative study in Tamil Nadu, India

**DOI:** 10.1186/s12889-021-10352-4

**Published:** 2021-02-12

**Authors:** Rajalakshmi RamPrakash, Lakshmi Lingam

**Affiliations:** 1 Loyola Institute of Business Administration, Loyola College Campus, Nungambakkam, Chennai, 600034 Tamil Nadu India; 2 Tata Institute of Social Sciences, V.N. Purav Marg, Deonar, Mumbai, 400088 India

**Keywords:** Gender, Publicly funded health insurance (PFHI), Universal health coverage (UHC), Social relations (SR) framework, India, Gender analysis

## Abstract

**Background:**

The continuing impetus for universal health coverage has given rise to publicly funded health insurance schemes in lower-middle income countries. However, there is insufficient understanding of how universal health coverage schemes impact gender equality and equity. This paper attempts to understand why utilization of a publicly funded health insurance scheme has been found to be lower among women compared to men in a southern Indian state. It aims to identify the gender barriers across various social institutions that thwart the policy objectives of providing financial protection and improved access to inpatient care for women.

**Methods:**

A qualitative study on the Chief Minister’s Comprehensive Health Insurance Scheme was carried out in urban and rural impoverished localities in Tamil Nadu, a southern state in India. Thirty-three women and 16 men who had a recent history of hospitalization and 14 stakeholders were purposefully interviewed. Transcribed interviews were content analyzed based on Naila Kabeer’s Social Relations Framework using gender as an analytical category.

**Results:**

While unpacking the navigation pathways of women to utilize publicly funded health insurance to access inpatient care, gender barriers are found operating at the household, community, and programmatic levels. Unpaid care work, financial dependence, mobility constraints, and gender norms emerged as the major gender-specific barriers arising from the household. Exclusions from insurance enrollment activities at the community level were mediated by a variety of social inequities. Market ideologies in insurance and health, combined with poor governance by State, resulted in out-of-pocket health expenditures, acute information asymmetry, selective availability of care, and poor acceptability. These gender barriers were found to be mediated by all four institutions—household, community, market, and State—resulting in lower utilization of the scheme by women.

**Conclusions:**

Health policies which aim to provide financial protection and improve access to healthcare services need to address gender as a crucial social determinant. A gender-blind health insurance can not only leave many pre-existing gender barriers unaddressed but also accentuate others. This paper stresses that universal health coverage policy and programs need to have an explicit focus on gender and other social determinants to promote access and equity.

**Supplementary Information:**

The online version contains supplementary material available at 10.1186/s12889-021-10352-4.

## Background

The need to achieve universal health coverage (UHC), “including financial risk protection, access to quality essential healthcare services, and access to safe, effective, quality, and affordable essential medicines and vaccines for all” has been incorporated in Target 3.8 of Sustainable Development Goals [1]. UHC is an important component of social protection mechanisms to manage health risks for the vulnerable populations so that health shocks experienced do not lead to a vicious cycle of debt, poverty, and ill health. It is estimated that in India alone, more than 63 million households fell into poverty within a year due to inability to finance out-of-pocket health expenditure [2]. The unequal gender power relations in distribution of work, property, and other resources have been described in feminist literature [3–6], but the extent to which public policies address gender as a health determinant is less understood [7]. Lower utilization of healthcare and lower expenditures for female hospitalizations observed in several studies in India [8–10] has reiterated continued existence of gender inequity in resource allocation and the need for policies to address this gap.

Health policy analyses often discusses health inequities in income or class irrespective of the gender of members, representing horizontal equity, that is, equal treatment of those with equal needs [11]. Gender equity, however, requires recognition of vertical equity, that is, different needs of men and women (that may or may not stem from biological differences) so that systematic differences are eliminated in terms of allocation of resources [12, 13]. The term “systematic” implies that some differences in health indicators based on characteristics such as gender, race, education, income, disability etc., are not random [14] but pervasive owing to the unequal social positions. In countries like India, religion and caste are important social axes that stratify society that have shown to affect healthcare access resulting in health inequities [15]. Yet they are avoidable, provided resources are allocated explicitly to eliminate them. The Commission on Social Determinants of Health observed that “this unequal distribution of health damaging experiences is not in any sense a ‘natural’ phenomenon but is the result of a toxic combination of poor social policies and programs, unfair economic arrangements, and bad politics” [16] p5.

In other words, where women are found to have higher rates of morbidity and hospitalization and also control fewer resources, the programs and policies of financial health protection in those areas need to aim specifically at women. A feminist approach to health inequities, especially in the area of public health, requires study of the linkages between “disadvantage and health, distribution of power in the processes of public health” [17]. Concerns have been raised that UHC schemes across countries that have not given explicit attention to women’s specific needs, importantly but not limited to sexual and reproductive health (SRH), could exacerbate gender inequities [18, 19].

Since 2007, both central and state governments^1^ in India have introduced several publicly funded health insurance schemes (PFHIS) for the poor and marginalized sections to fulfill UHC objectives. A literature review on PFHIS in India in 2018 [20] points that most studies used households (low-income, rural, and caste) as basic units of analysis and focused on economic elements. This left gender as an under researched dimension. Lack of clear understanding of the complexity of gender and other intersecting social determinants, combined with failure to apply appropriate gender evaluation frameworks, had limited the focus on only maternal health and hysterectomies in these studies.

While much has been discussed about gender inequities in accessing health services at household and community levels, there is hardly any research on health services provided through the insurance route, certainly not those that look at gender-based biases in policy design, administration, or market influences. To our knowledge, this is one of the few papers that comprehensively discusses all aspects of a public policy that involves public and private stakeholders using a gender lens in a way that unearths complex realities as experienced by marginalized end users.

The focus of this paper is a gender analysis of the Chief Minister’s Comprehensive Health Insurance Scheme (CMCHIS) implemented in the southern state of Tamil Nadu (TN), India. There is little literature on this scheme [21–23] and none on the gender dimensions.

According to a study based on the Government of India’s nationwide household survey data [24], women in TN had a higher self-reported morbidity and higher hospitalization rate than men [25]. Since all members in the household, irrespective of gender, were eligible to access the CMCHIS, it was expected that women would make use of the scheme to avail the financial protection to access inpatient care. On the contrary, data from insurance claims in the TN scheme point to gender inequity in utilization by women with higher share for males in number and value of claims [26]. The pro-male bias has also been found in utilization of PFHIS in other Indian states [20]. In fact, in TN, a decline in the share of female beneficiaries in total claims was observed, from 39.7% (in 2012–13) to 34.6% (in 2015–16), even when the overall claims increased [21]. These findings raise serious concerns on the gender equity implications of allocation of health resources of the CMCHIS.

The Research Questions (RQ) addressed by this paper are:

- RQ1: What are the gender-based barriers that women face to access inpatient healthcare services under the CMCHIS?
- RQ2: Where are these gender-based barriers located—in the micro sphere (household), meso sphere (community and local health systems), or macro sphere (market, State health systems, policies)?

This paper aims to answer the above questions drawing from the qualitative arm of a mixed-methods study.^2^ The data generated from in-depth interviews with men and women who were hospitalized (insured and uninsured under the CMCHIS) and stakeholders (public and private health providers, CMCHIS administrators, health activists), as well as field notes during research have been systematically analyzed using the gender analysis framework developed by Naila Kabeer [27] called the Social Relations (SR) Approach/Framework.^3^

The SR framework is chosen specifically because it unravels gender-based inequalities not only in micro and meso spheres of household and community but also in macro institutions of State and market. Using the findings of the study, we argue that unequal gender relations are mediated as a social determinant, not only in the household or community levels but in the way the market and State dictate policy design and implementation of a specific PFHIS. We also highlight how lack of a gender lens in PFHIS can accentuate gender-based barriers for women and work against gender and health equity.

Beyond this introduction, the paper is structured as follows: The setting and methods employed in the study and operationalization of the SR Framework are described with supporting information in the first section. The results section consists of the gender-based barriers arising out of the institutional analysis of the household, community, market, and State. We unpack each institution in terms of the rules, activities, resources, people, and power dimensions and their outcomes on access to healthcare through the CMCHIS. The discussion session brings together how each institution reinforces gender biases. Implications for policy and research is given before concluding the paper.

## Methods

### Setting

TN is the 11th largest state in India by area (similar to Iran) and 7th most populous in India with a population of 79.3 million [30] divided into 32^4^ districts. TN is one of the highly developed states in India [23], with approximately 11.28% (compared to 21.92% the rest of India) people falling below the poverty line: 15.8% in rural and 6.6% in urban [31, 32]. Although known for its robust public health system, the private health sector is a dominant player in TN, with a 65.4% share in all inpatient episodes. In 2014, its people spent on healthcare an average amount that is 12 times more in private sector compared to the public sector [21]. During the time of data collection, i.e., 2017, the TN government had not published the Rules for the Tamil Nadu Clinical Establishments (Regulation) Act of 1997, which aimed to register and regulate the functioning of both public and private health establishments.^5^ The lack of regulation has particular relevance to the behavior of healthcare providers, barriers to access healthcare, financial protection and grievance redressal under the CMCHIS.

#### The CMCHIS

The CMCHIS, introduced in 2012, aimed “to provide free medical and surgical treatment in Government and private hospitals to the members of any family whose annual family income is less than INR 72000/- (One USD approximately 70 INR) per annum”.^6^ It was expected to cover low-income households in TN and enroll all members of the household irrespective of sex, age, and pre-existing health conditions. The main documentary proof of eligibility is the ration card along with an income certificate issued by the local revenue officer. The ration card is an important document which entitles the listed household members to avail free or subsidized essential commodities and also serves as an identity document for receiving other welfare benefits, including the insurance scheme.^7^ While according to the Government of India sample study, only 17.8% of the TN sample households were enrolled in any form of government-sponsored insurance scheme [24], the TN government claimed that 55% (42 million individuals) were enrolled in the CMCHIS [30].

The CMCHIS is based on a public–private partnership (PPP) model, where there is a separation of the financing from the purchasing of services. The State is the public partner and is also the overall governing agency. The contracted insurer pays and purchases health services via third party administrators (TPA). Private and public hospitals that get formally included as an empaneled network hospital under the CMCHIS provide health services. In the CMCHIS, the insurance company, along with the TPAs, undertake awareness generation, enrollment, facilitating eligible cases to get coverage, and perform claims management [21, 26]. The insurer receives the premium from the government for every enrolled household and reimburses the cost of services provided by the hospitals (private and public). The assumption in such a model is that such schemes will lead to expansion of private hospitals in underserved locations, provide “free choice” to patients to choose between different healthcare providers and “money follows the patient” [26].

### Research design

For the doctoral study carried out by the first author, one urban district (Chennai) and one rural district (Salem) were selected based on socio-economic indicators. Within each district, a low-income locality was chosen based on estimate of hospitalization rates, eligibility to enroll in the CMCHIS, and expected utilization of the CMCHIS. However, the districts differed in the type and geographical distances of healthcare facilities. Names of the specific localities are not disclosed to maintain confidentiality. The urban study site was characterized by thatched houses, poor drinking water, and sanitation facilities with a mixed religious population engaged in informal occupations. Public and private hospitals providing inpatient care were available within 3 kms from the study site. The rural study site was hilly, where most households lacked toilets, and members were mostly Hindu, landless, or small landholders engaged in rain fed crop cultivation. The closest facilities providing inpatient care (other than maternity) were 17 kms (public) and 55 kms (private) from the study sites.

The primary study followed a sequential mixed-methods design with qualitative arm following the quantitative arm [33]. The quantitative arm included a house listing of each and every household in the demarcated locality resulting in a total of 1176 households (640 in the urban and 536 in rural). Besides the sociodemographic and CMCHIS enrollment details, the hospitalization details of all family members of the last three years were captured during the house listing. From this, a list of those with at least one hospitalization episode in the reference period were considered eligible as in-depth interview respondents.

### Selection of respondents

For the reference years 2014 to 2016, a total of 76 women with a history of hospitalization (54 in urban and 22 in rural) were identified from the house-to-house survey, out of which 17 (13 in urban and 4 in rural) were children below 18 years, hence excluded from the study. Each person identified through survey was contacted by the first author at her/his home and details were given about the nature of qualitative research (aim, time required, expected results, risks, and benefits) with a printed Participant Information Sheet in local language. Sufficient time was given to answer queries and a day was fixed for those individuals who expressed willingness for an interview. Out of the remaining 59 women, three women were not eligible to consent (one was mentally challenged and two were still recovering from surgery). Three women could not be contacted (they moved from the address given during the survey or were not available in their homes in spite of being visited three times). Seven women refused consent and as per the ethical protocol of the study it was not mandatory for them to state the reasons for their refusal.

Among the women that consented to be interviewed, the details of the women were categorized based on

(i) whether they were enrolled in the CMCHIS and whether they utilized the CMCHIS
(ii) type of facility for most recent hospitalizations (public or private)
(iii) type of ailment leading to most recent hospitalization (chronic: cardiovascular, musculoskeletal, neurological, and cancers; acute: infectious)
(iv) sociodemographic background (age, location, religion, caste, marital status)

It was decided that selection of respondents should ensure that at least two respondents of each sub-category is chosen to ensure diversity and richness to the data. Narrative interviewing technique [34] was employed and interviews were held in respondents’ home and lasted between 60 and 90 min. Respondents freely narrated their health histories up to the recent hospitalization and were followed up with guidelines (see Additional file 5) to get additional details. After interviewing 33 women, data saturation was achieved in patterns of household support, treatment seeking, enrollment, client–provider interaction, hospitalization experience, and health expenditure support. As sufficient quality (rich) and quantity (thick) of data [35] were attained and no new patterns were emerging [36], further interviews were stopped.

In order to understand the barriers experienced by males, 86 males with history of hospitalization were categorized in a similar way. However, due to lack of availability of male interviewees during day times and safety concerns of the unaccompanied female researcher, only 16 in-depth interviews were completed with men. All in-depth interviews were conducted in local language (Tamil) by first author at their homes. Table 1 represents the selection process of respondents for in-depth interviews.

**Table 1:** Selection of In-Depth Interview Respondents

***Filter Criterion***	***Urban District***		***Rural District***		***Total***	
	Female	Male	Female	Male	Female	Male
Hospitalized at least once in last three years	54	57	22	29	76	86
Only those above 18 years	41	39	18	26	59	65
Available at residence within three attempts	38	18	18	16	56	34
Eligible to consent	36	18	17	16	53	34
Explained about research and consented	29	10	17	7	46	17
Finally interviewed	16	9	17	7	33	16

For the 14 stakeholder interviews, respondents were selected purposely based on their roles and experience with the CMCHIS. Stakeholders within the study sites included village administrative officers (VAO), public and private health providers, liaison officers in hospitals, hospital administrators, and health activists. Three stakeholder interviews from the implementing agency (Tamil Nadu Health System Project) and insurers were planned but only one interview could be conducted because of transfers and lack of cooperation. All stakeholder interviews were done by first author (all except 3 in Tamil) primarily to understand the administrative aspects of the scheme and triangulate the findings from in-depth interviews. The entire data collection in both districts took place between February and July 2017.

The number and category of in-depth interview and stakeholder interview respondents are summarized in Table 2 and Table 3, respectively.

**Table 2:** Summary of In-Depth Interview Respondents

	***Utilized CMCHIS***	***Not Utilized CMCHIS***		***Total***
		Enrolled	Not Enrolled	
Urban Women	5	2	9	16
Rural Women	6	9	2	17
***Women (Total)***	**11**	**11**	**11**	**33**
Urban Men	4	4	1	9
Rural Men	2	4	1	7
***Men (Total)***	**6**	**8**	**2**	**16**
***Total (Men and Women)***	**17**	**19**	**13**	**49**

**Table 3:** Summary of Stakeholder Interview Respondents

***Stake Holders Interviewed***	***Urban District***	***Rural District***	***Total***
Village Administrative Officers	0	3	3
Private Healthcare Providers	2	3	5
Public Healthcare Providers	1	2	3
Implementing Agency representative	1	0	1
Healthcare activists	1	1	2
TOTAL	5	9	14

Background characteristics of in-depth interview respondents are given in Additional file 2 and Additional file 3.

The in-depth interview guidelines covered general health history, nature of paid and unpaid work, healthcare-seeking patterns, awareness and experiences with enrolment in the CMCHIS, decision-making regarding recent hospitalization and utilization of the CMCHIS, client–provider interactions, health expenditures, quality of care, perceptions of the CMCHIS, and current health conditions and challenges. Stakeholder interviews included respondents’ role in the CMCHIS, eligibility verification, preauthorization and claims process, deciding to provide coverage under the CMCHIS, perception on the impact of the scheme including equity issues and challenges, and any cross validation of other findings.

On the day of the interview, the first author cross-verified if the participant had understood the key elements of research and procured signatures on the informed consent sheet prior to the interview. Written consent for audio recording was procured separately. Since some respondents could not read, the contents of the form were read out in the presence of a literate family member who signed on their behalf. Some stakeholders did not consent for audio recording, citing privacy reasons, and only oral consent was obtained. All the recordings were transcribed and translated into English by the first author before analysis was done. The first author maintained a field journal, where key observations during the interviews, such as non-verbal communication, questions asked to the researcher, and interaction between family members, were recorded.

The research proposal, data collection tools used for in-depth interviews, and stakeholder interviews, including the participation information sheet and informed consent forms, were drafted in the local language and English and were approved by the Institutional Review Board (IRB) of Tata Institute of Social Sciences, Mumbai, India.

### Social Relations (SR) Framework

Among the many gender analytical frameworks that have been developed since the 1980s to inform development practice in order to instill gender sensitivity [37], Kabeer’s framework recognizes power, hierarchy, and inequalities embedded in institutions like the household, community, market, and State in governing gender relations. The earliest form of this framework is seen in Kabeer’s book *Reversed Realities: Gender Hierarchies in Development Thought* (1994) [27]. Later publications by Kabeer and Subrahmanian in 1996 [38] and 1999 [39] have elaborated the SR Framework further. Kabeer’s ideas are influenced by socialist feminist perspectives that place emphasis on understanding the linkages between production and reproduction, between capitalism and patriarchy, and between the economic and the cultural. Attempts to empower women and achieve gender equality and development goals have to cover the ground of the micro and the macro.

The five dimensions of institutional relationships that require to be unpacked, according to Kabeer (1999) are: *rules*, or how things get done which may be written or unwritten, formal or informal; *activities*, or who does what, who gets what, and who can claim what; *resources*, or what is used and what is produced, including human, material, and intangible resources; *people*, or who does what, assignment of resources, responsibilities and hierarchies, or inclusions and exclusions; and *power*, or who decides and whose interests are served. These dimensions dictate the internal dynamics of the institution that goes beyond the supposed expectation from that institution [27]. The framework alerts us to unequal SR, which in turn determine differential access to resources, responsibilities, and power creating a variety of barriers to men and women, but more intensified for women and girls [28, 29].

**Fig. 1:**
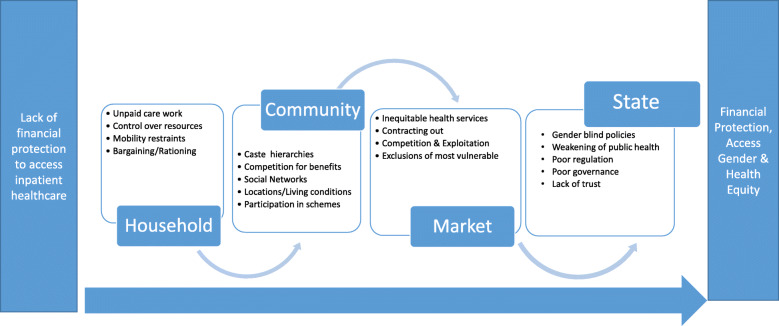
Framework for Gender Analysis of PFHIS. *(Source: Authors)*

For this paper, the SR Framework was considered as important and operationalized in the following manner. An access to healthcare pathway was hypothesized as a continuum with lack of financial protection to access inpatient treatment on one end of the spectrum and gaining equitable access to healthcare at the other end of the spectrum, made possible by the CMCHIS. The transition from one end to the other end is linked to key aspects like design of the scheme, awareness generation, enrollment of clients, client–provider interaction in the healthcare setting, and actual utilization. The four institutions as mentioned in the SR Framework—household, community, market, and State—and their dimensions—rules, activities, resources, people, and power–intervene at all the stages of this continuum. The SR operating within and across the institutions on these dimensions give rise to three types of gender barriers: barriers that are specific to each gender (gender specific); ones that are common but felt more acutely by women (gender intensified); or ones that are covertly translated into norms and practices (gender imposed). The authors acknowledge that operationalizing the framework for the study of gender dimensions in the CMCHIS was cumbersome, with considerable overlaps in institutions, dimensions, and resulting gender barriers. However, for simplicity and clarity, they have been represented as in the Fig. 1.

After translating the interview transcripts to English, they were imported to Atlas Ti v7, and then analyzed line by line. Both inductive and deductive reasoning were applied for content analyses. Initially, open codes emerged iteratively during reading of transcripts using inductive approach, for example, continuous domestic work, discretion of providers, denying low package, selective information, domicile change with marriage, etc. Deductive approach helped to assigning the codes to categories such as whether the experience was gender specific, intensified or imposed, and whether these were located in household, community, market, or State. Within each institution, rule, activity, resource, people, and power were coded along with the stage of the scheme cycle (design/awareness/enrollment/utilization/impact) during which these dynamics were experienced. These helped to identify overall themes such as double burden of unpaid care work, rationing of healthcare, gender blindness of policy, etc. The transcripts with the codes were reread several times, shared between authors, and categories assigned were cross-verified for validity [40].

## Results

Based on the 33 in depth interviews with women, 16 with men, and 14 stakeholder interviews, study results are presented below. A summary of codes, subcodes, categories, and themes that emerged from the study are given in Table 4 (refer to Additional file 1).

In the following section, results are presented to answer what the gender-based barriers (RQ1) by identifying the rules, activities, resources, people, and power distribution are and how their interplay gives rise to one of the three types of gender barriers. A summary of all gender-based barriers is given in Table 5.

**Table 5:** Gender Barriers in the CMCHIS [Source: Authors]

***Gender Specific***	***Gender Intensified***	***Gender Imposed***
(Barriers that are closely linked to masculinity and femininity norms, exclusive to each gender)	(Barriers faced by both men and women but intensified for women)	(Gender biases reflected in institutional norms [written or unwritten])
***For Women:***		
Restricted mobility to participate in community activities and reach healthcare services	Lack of literacy and poor participation in awareness and enrollment activities of scheme	Requirement of female attendants for hospital admissions for women
Continuous burden of care work (cooking, cleaning, caring for old, young, sick)	Lack of documentary evidence to access scheme benefits	Insurance policies discriminating women who are not wives or mothers or outside mainstream relationships
Notions of sacrifice and guilt	Age, marital status, class, caste, political inequalities intersect with gender to accentuate barriers	Impact of health insurance measured with household as an egalitarian unit making women’s experiences invisible
Poor perception of one’s own health needs	Poor negotiating skills with persons of authority to claim entitlements	
***For Men:***		
Increased mobility resulting in risks of accidents	Cherry picking by providers and induced utilization	
Risk taking and aggression resulting in perpetrating and subject of violence	Poor financial protection from OOPE resulting in delay in hospitalization and distress coping	
Burden of bringing income as head of household	Insurance mechanisms weakening public healthcare institutions	

The stage of the scheme cycle where they most impact is also matched. This section also answers where these barriers operate (RQ2) by placing them under the relevant institution (household, community, market, and State), though these are not water-tight compartments. Under each institution, we provide quotations and case-lets (in italics) to provide evidence for the activities, resources utilized, and which members are involved that mediate the rules and unequal power relations.

While discussing results in this paper, all respondents have been given pseudonyms, location (urban or rural), and caste (Scheduled Caste [SC] or Other Backward Caste^8^). Further details such as religion, age, and stakeholder’s affiliations have been hidden in order to fully protect and ensure the anonymity of the respondents. The authors have ensured that the paper complies with the Standards for Reporting Qualitative Research (SRQR).^9^

Table 6 (given as Additional file 4) describes the five dimensions interplaying within each of the four institutions, type of gender barriers, and implications for access to healthcare using the CMCHIS.

### Gender barriers mediated by households

Households of most interviewees typically consisted of an earning male member who was also the head of the household, as reflected in ration card and the CMCHIS card. The income-earning male controlled the financial and non-financial resources and took major household decisions. The women were responsible for care work and household chores and needed to consult others to take time or money for travel or other needs. The continuous and uninterrupted demand of a woman’s care work combined with lack of control over resources affected decision-making around her health.

The narratives in this study revealed that even when engaged in paid work, women respondents did not enjoy autonomy to take their own healthcare decisions and for financing them. A woman’s mobility constraints, burden of care work at home, negotiating power and availability of substitutes for care work, and level of internalization of gender norms played a role in determining whether and how early she sought healthcare treatment. Based on other intersectional positions such as age, marital status, disability status, within the home, the woman’s vulnerabilities and dependencies were accentuated.

*I have been telling my sons to take me (to the hospital) … however, where do they listen? They say they have this work and that … then I decided to go myself. I did not know which bus and where to get down … then some people guided me ... gathering courage finally I went one day.*

*(Kanchana,*^10^*Rural, Other Backward Caste)*

Lack of a female member in the family circle to provide backup for carrying out domestic work for women resulted in poorer bargaining position within the household and also vis-a-vis the health systems.

*The chief doctor also told me that after the operation I could go home in 3 days. But my husband told the doctor that there is no one at home except me to take care of him and the kids ... and requested the doctor to discharge me. The doctor also said that it was just a matter of three days, but my husband asked to discharge me. [pause] If I had the operation earlier, my lungs would not have got affected like this … that is what I keep telling my husband*.

*(Sulaima, Urban, Other Backward Caste)*

Women’s narratives point to strict gender roles, where women are expected to be solely responsible for all the domestic work such as cooking, cleaning, washing, and caring for the old, young, and sick. Many women reported that men and boys cannot be expected to take up domestic and care work and even felt guilty asking for support from them when they were sick or hospitalized. The narratives overall point to the enormity of the burden of women’s care giving work within the household which created barriers to hospitalization. According to the Organization for Economic Cooperation and Development (OECD), Indian women spend up to 352 min per day on domestic work, 577% more than men (who spend only 52 min).^11^

In-depth interviews showed that even when not explicitly told to, many women preferred to tolerate and remain in silence until the illnesses became very serious. For instance, the case of Begum below points out how internalized gender norms, combined with poor awareness, prevented early diagnosis and instead resulted in hospitalizations for surgical interventions.

Begum had heavy uterine bleeding for more than four years and was advised to undergo hysterectomy by the doctors in the public hospital. When asked why she kept delaying, she said:

*I was feeling bad that there is no one to cook and serve food for my husband and my son, that is why I did not get admitted. There is no one to take care of the family as my daughter is married and gone.*

*(Begum, Urban, Other Backward Caste)*

In contrast, men’s illnesses were hardly shrouded in silence and were attended to relatively sooner depending on the financial situation. Methods of financing and coping with out-of-pocket health expenditures differed across different family members based on gender, age, and marital status. Due to limited participation in paid work, women were dependent on the male members for even financing their outpatient consultations.

*My husband said (in a slightly angry tone), “How much can I spend? Now only you spent 500 (INR). You were eating and doing okay only no? ... again another doctor now ... 500 here, 500 there ... Frequently you are spending 500 rupees.” ... I was thinking about this. So without telling anyone I went and got admitted in a government hospital. Instead of being pointed out again and again, I myself went and got admitted, got better, and came back.*

*(Rahima, Urban, Other Backward Caste)*

Findings reveal that resources are more quickly mobilized for men’s hospitalizations in the form of loans and sale of assets. Admissions for men are mostly in the private hospitals because of perceived better quality of care and quicker recovery. As gender-disaggregated data on health expenditures in India [5] show, the interviews too revealed that the household took men’s hospitalizations more seriously as they were considered as breadwinners. Some women even said they would ration the benefits of the CMCHIS card for their children or spouses.

*Participant: “I will keep it (CMCHIS card) for them.”*

*Researcher: “What about you?”*

*Participant: “I can go to government hospital. The card can be used in private hospitals.”*

*(Selvi, Urban, SC)*

Women themselves believed that all household resources, such as cash and assets including health insurance benefits, were meant to fulfill men’s (also children’s) needs and their own needs cannot be a priority.

The following excerpts from the same respondent, the first one showing the choice of facility for women’s hospitalization and the second one for men’s hospitalizations, contrast the decision-making process:

*If we save that (hospitalization) money, it will come of use for the family ... Instead of giving in their (private hospital) hands for us to get better, if we go to government hospital, we can save it and use to repay a loan or buy for the house ... That time ... these things will definitely run in our (every woman’s) mind.*

*Somehow they [men] should become okay. Even if we have to lose all the things. Even if we have to sell the jewels. Their health should be okay. If they are okay only, they can take care of us, right? If they become okay only, we will have respect in society. I think like that.*

*(Nalini, Urban, Other Backward Caste)*

In-depth interviews revealed that while loans were taken for men’s hospitalizations, women’s hospitalizations relied on contributions from maternal home, employers, close family members, or sale of women’s meagre assets.

*Begum had to spend INR 85,000 for her hysterectomy by borrowing from the woman employer where she worked as a cook. As it was not enough, her married daughter gave back jewelry that she was gifted earlier, which was then sold to finance the medical expenditures.*

*(Begum, Urban, Other Backward Caste)*

Women like Anandhi had to sell off their cattle or jewels or like Lingamma, discontinue their daughters’ education to finance or support if any of the family members needed to undergo treatment.

The narratives of in-depth interviews revealed how different social positions (gender, class, urban/rural location/caste, age) intersected to determine access to healthcare treatments. For instance, though an illness in men, especially those who were breadwinners, received earlier attention compared to women within a household, the decision to hospitalize and undergo a surgery was delayed if the man belonged to a very poor family. This was due to fear of loss of income even for a temporary period during hospitalization and insecurities of returning to work post-surgery. Elderly, differently abled, unmarried, and deserted women were found to have stronger feelings of guilt for using household resources for their own healthcare compared to other women. Such women were also less likely to own documentary proof required to access scheme benefits. Also, while private hospitals were preferred for men’s hospitalizations in general, in a well-off family, even women preferred to seek private treatment. The very poor men and women sought care in public hospitals as private hospitals were unaffordable to them.

Some women interviewees did not live in typical male-headed households and had additional barriers to access the scheme benefits. These included a transwoman who left her home, a sex worker, second wives,^12^ domestic violence survivors, and women who were separated. Such women did not possess ration cards with their names listed or had access to them. Within a “regular” household, the unmarried, differently-abled, and elderly widowed women felt they were a burden to the family and did not prefer to claim benefits from the scheme.

*Kamakshi lived in a two-by-five feet tinned house in an urban slum with two children. She had run away from her marital home as her husband and mother–in-law abused her physically and mentally. She was recently hospitalized for stomach ulcer in a public hospital and, on probing, was found to suffer from anemia and hypertension as well. When asked about enrolling in the CMCHIS card, she said, “I went to Collector office (kiosk*^13^*) to apply for card, but they are asking for ration card, caste certificate, this and that ... Even my husband doesn’t have ration card because his mother was a second wife herself.”*

*(Kamakshi, Urban, SC)*

### Gender barriers mediated by communities

The study found that community power structures based on social (class/caste) grouping influenced the access of residents to information and participation in the CMCHIS. The urban community was organized on the lines of religion^14^ and class, whereby the wealthier among them had houses with concrete roofs, independent toilets, closer to main road locations, and access to drinking water. The poorest households were *kuchcha*^15^ houses, situated along the drainage ways and railway tracks with no access to drinking water and toilets. Though Muslims lived close to each other, they were found both in well-off sections and impoverished sections of the urban study site. The rural community was primarily Hindu and organized on caste (and occupation) lines with members (usually landless) of lower caste settled together in segregated settlements, often referred to as “colonies”.

Interviews revealed that the location of households mediated their access to information and participation in activities, including those related to the CMCHIS. The more remote locations are usually occupied by the impoverished, stigmatized, and marginalized groups, leading to greater levels of social exclusion and deprivation.

In urban areas, women in households near the railway tracks (farthest from the main road) complained that many times, information about welfare activities never reached them because “others” did not want them to benefit.

*Participant: Many have taken [card], [hand pointing to the streets near main road] … For us only a few of us have it, half of us don’t have the card.*

*Researcher: Why did they leave out this side?*

*Participant: They are leaving us because those in the opposite side, they themselves will tell to outsiders that there are no people living this side here.... Those women are “gaandu” [vengeful] …*. *They think they only should benefit.... If we confront them, they will fight.... That is why they don’t come inside much.*

*(Shehnaz, Urban, Other Backward Caste)*

In rural areas, interviews indicated a clear stigmatization of the SCs, for example, entering the “colonies” was considered as lowering the status of the other (dominant) caste members, even for health providers and researchers.

*If there is anything to say or give, we don’t go there [to SC colonies], we send word to them to come here [under the tree near Panchayat office]. We do everything from here itself*.

*(Stakeholder Interview, Rural)*

The rules of who can enter where in the geographical community in fact affected the data collection process as well, where the dominant caste survey volunteers refused to enter the areas occupied by SCs and even prevented the first author from entering those “stigmatized” areas. The urban and rural pockets occupied by SCs were generally perceived as “unsafe” by the rest of the community that conjured up ideas that drug trafficking, prostitution, and other illegal activities take place in these parts of the neighborhood. This process of “othering” built a narrative to justify the distancing and exclusion of these marginalized communities.

Meetings, awareness programs, or enrollment camps were usually conducted in a school or marriage hall located in the dominant part of community. Given that caste segregation was practiced in the community, it is possible that many from the “colonies” or from railway track areas did not attend it. Interviews with stakeholders revealed that no specific measures were taken to cover the lower-caste habitations.

One of the village functionaries entrusted with the CMCHIS awareness generation, when asked about the awareness activities said, “*We covered the entire village by going in an autorickshaw and making announcements.*” However, the researcher found some hamlets in the village were not accessible by an autorickshaw.

Some women reported that the information about camps came at short notice and they could not set aside time, effort, and money to travel from their homes to the enrollment camps and hence decided to miss them.

*When they gave it, I didn’t go madam. I didn’t know, no one told. [When told] I also had a lot of work at home ... fetch water, cook, wash clothes, have to go for function, to the meat shop, after that, have to see whether there are tomatoes and onions at home, then have to buy, then check if there is oil …. I just left it. If there is something important, we have to leave all this aside and run.*

*(Rahima, Urban, Other Backward Caste)*

Another way in which the community mediated access was through the authority figures and street-level bureaucrats. Street-level bureaucrats, a concept introduced by Lipsky [41] refers to those public functionaries who interact with citizens at the ground level and who use their power to influence the access to benefits promised under policy. In the study, those who had acquaintances with these key people in the community who wield power, such as political party members, VAOs, ration shop owners, hospital staff, and district kiosk staff, found it easier to access welfare scheme benefits including the CMCHIS.

*Last time the cards were issued by [panchayat] members ... but later it was found that they are giving to those known to them only …. It was found here and there ... so this time all instructions came that the cards should be with the VAO only.... We have distributed as much as possible.*

*(Stakeholder Interview, Rural)*

Begum and few other women respondents reported that they obtained the card even without going to any enrollment camp because their husbands knew the political party members. Some women (especially single, unmarried, differently abled) felt they had limited mobility and did not have the information or the connections with key people in the community to get access to schemes. For example, Mary, who was separated from her husband, reported that the ration shop owner in her area did not allow her to include her son’s name because she was separated from her husband (thus suspecting the son’s paternity). Manjula, who was the second (not legally sanctioned) wife of a village man, decided to miss the enrollment camp because she did not want to be embarrassed when the authorities enquire who was the head of the household (her husband had already enrolled in the CMCHIS with his first wife). She also said that her name was struck off in her paternal household ration card and she is not sure if she would get a new ration card.

While all women in general participated less in awareness generation activities of the CMCHIS than men, the SC women who lived in segregated pockets in the community were further deprived. Besides, the continuous domestic work put women at a disadvantage compared to men, even if they belonged to the same “colony”. While women in non-male-headed household had autonomy in household decisions, they found it difficult to establish and maintain friendly relations with authority figures and to negotiate bureaucratic processes to obtain the CMCHIS benefits. Thus, unequal distribution of activities, rules, and resources sustained gender-intensified barriers in the community.

Another important finding was that the local self-help groups and non-government organizations in the study areas were not involved in the design, implementation, or monitoring of the CMCHIS. Lack of community participation in government-sponsored health insurance schemes has also been highlighted in another study in TN [21].

### Gender barriers mediated by market

In this section, we present results that focus on how the health (public and private) and insurance systems operating with market ideologies of profits, choice, and competitions have changed the way healthcare services are delivered. The experiences of men and women of low-income households while attempting to access healthcare through the CMCHIS, triangulated by stakeholders, speak to a range of design level and implementation exclusions, denial, and delay in care, imposing conditionality, out-of-pocket expenditures (OOPEs), superficial awareness, and enrollment activities, all of which accentuated disadvantages for women’s health, especially the most vulnerable.

Even though the CMCHIS covered a range of procedures, by design, most of them were high-end surgical procedures indicated as packages, which can be done only in tertiary-level hospitals. In fact, the share of high-end packages from cardiology, cardio-thoracic, and orthopedics in the overall claims from the CMCHIS was reported to be 52.4% according to one study [26]. Private hospitals preferred to select and admit patients who required such treatment compared to procedures with lower packages, irrespective of the fact that for poor patients, even mobilizing such small amounts was difficult. For instance, many women in the interviews reported that they were told by providers at private hospitals that the hospital can cover only treatments which are extremely costly, often expressed as “operations above one lakh rupees”.

*I asked if this [surgical correction of fracture] will come under the CMCHIS, they [private hospital] said ... this is only a small surgery … it will only cost INR 20,000–30,000 they said …. I came back.*

*(Anandhi, Rural, SC)*

The CMCHIS, similar to private health insurance, did not cover outpatient consultations, cost of drugs, and attendant and transportation costs, which formed a major proportion of health expenditures causing considerable financial burden for lower-income women, as we saw in the case of Rahima.

Narratives revealed that profit motives of private providers influenced the quality of information shared with patients on their entitlements under the scheme and even resulting in taking unnecessary cash advances and OOPEs.

*I asked [private hospital doctor] how much will it cost with you, that is, without the card? He said INR 50,000.... I said sir that means I don’t have that much money sir, I may have to go to government [hospital]. He said that is your choice. So I asked will you use [CMCHIS] card? He said he will use. So I said please use the card. Then he said medicines I have to buy outside. That card will be for only operation, but for bed charge and for tablets and medicines, you have to give money and buy he said. So okay I said.... So totally INR 15,000 I spent. They didn’t say how much they have taken from the card and other details*.

*(Nandini, Urban, Other Backward Caste)*

The same market-based insurance principles allowed coverage for only low probability and high-cost illness events, and SRH services [42] were excluded from the CMCHIS. SRH ailments were found among 11 study respondents included malignant and non-malignant breast tumors, cervical cancers, spontaneous or induced abortions, urinary infections, infertility, need for contraceptive services, adolescent health concerns, and gender-based violence. However, except for cancer treatments and hysterectomies, others were not covered under the CMCHIS. Almost half of the women interviewees reported that they were not aware that they had diabetes or hypertension until it was diagnosed during their recent hospitalization. Stakeholders pointed out that while higher budgets were allocated for the CMCHIS, which provided high-end treatments, attention to primary care, to screen, identify, or treat early onset of the same illnesses, were inadequate.

Stakeholder interviews reveal that hospitals and providers feel that as insurance is projected as a way to earn revenue, it made hospitals compete with each other to maximize their revenues by increasing the higher-end insurance cases. This competition took place not only among different private hospitals but also between public vis-à-vis private and between two or more public hospitals. While corporate hospitals could invest in specialist doctors, infrastructure, and sophisticated equipment, the medium-sized private hospitals and certain levels of public hospitals are unable to perform high-end procedures, and thus missed out on generating better revenues.

The following interview excerpt shows the competition between tertiary- and district-level public hospitals in revenue generation:

*It is okay [to raise revenue through the CMCHIS] for the teaching [medical college] hospitals but here [secondary-level public hospital], we don’t have specialists. Only Dr. K, an orthopedic, is here.*

*(Stakeholder Interview, Rural, Public Hospital)*

Owners of small hospitals in the rural study site also expressed that they were unable to join the CMCHIS because they could not invest in high-end technologies. This, to a large extent, limited the availability of empaneled hospitals, left the public facilities to fend for themselves, and limited the “choices” for the rural poor.

Public providers also admitted that the pressure to meet insurance targets led them to using cajoling to coercing techniques to get patients to utilize their CMCHIS card for a treatment they expected to receive without a card:

*Here [community] most are [company] union people, they ask lot of questions [when asked to bring the CMCHIS card] .... We try to “counsel” them ... “convince” them.... Sometimes we say then only this hospital will get some funds to repair some machine and improve something … that treatment will get delayed otherwise. Sometimes this will change their mind, and they will agree.*

*(Stakeholder interview, Rural, Public Hospital)*

Nancy’s case explains how introduction of the CMCHIS in public hospitals has resulted in providing healthcare only for insurance-covered illnesses.

*Nancy, living in an urban slum, approached a nearby medical college public hospital multiple times for a lump in one of her breasts. Every time she was told that it was not cancerous and does not require a surgical removal. When the pain from her lump affected her tailoring work, she again approached the hospital and pleaded for removal of the lump. Even though she was given a bed, Nancy reported that she was suddenly discharged from the hospital without a surgery. She had overheard a conversation among the doctors that her surgery would not be an “insurance case”.*

*(Nancy, Urban, Other Backward Caste)*

Another effect of insurance targets in public hospitals was in expecting scheme cards as condition to receive treatment. The respondents often did not know how to negotiate with the providers or street-level bureaucrats. When “uninsured” patients approached public hospitals with “covered illness”, providers compelled poor patients to enroll in the scheme (through district kiosks) and return to get treatment, resulting in provider-induced insurance utilization. This was reported by both men and women respondents who underwent undue stress, faced income loss, spent additionally for transport and commissions, besides delay in treatment.

*They had to do heart surgery for me. They said go and get the card. My son went once or twice but he couldn’t get [card] …. Then I myself went there [to district kiosk] ... even though I was unwell.... Then luckily that day, one officer noticed me. I was feeling breathless ... then he asked for what I have come. I told him that the doctor told me to get the CMCHIS card. He immediately told the staff there … so I got it done*.

*(Kannan, Urban, SC)*

The insurance industry, along with its outsourced agencies such as the TPAs, also played on profit principles. The study found that insurers carried out poor awareness generation and shallow enrollment activities and attested to gender-insensitive policy norms. Almost all women respondents reported absent or minimal awareness generation activities in their localities. These activities were conducted by insurance company, their TPAs, or the vendors who arbitrarily selected the localities. Even in enrollment, the interviewees reported many gaps. Some said that in spite of having attended the enrollment camp, they did not receive their CMCHIS cards. This was verified by a scheme administrator who stated that the insurance company did not adequately capture all household details (names of all members, complete address) and failed to distribute the cards, resulting in more than 350 undistributed cards (more than 60% of sample households) lying in the office (also verified by first author in a visit).

As in commercial insurance, where one card is given per family, the CMCHIS also followed the rule of one scheme card issued for every household with the name of the head of the household. During the interviews, women said they misunderstood that only the head of the household (husband) is eligible to use the card. The language of the CMCHIS policy was borrowed from private health insurance policies whose definitions of “employed” person, “legal” spouse, and “dependents” did not match with multiple forms of living arrangements of around 13 women interviewed.

For example, according to the CMCHIS website:

*A family is defined as one which includes the eligible member and the members of his or her family as detailed as below:*

*(i) Legal Spouse of the eligible person.*

*(ii) Children of the eligible person.*

*(iii) Dependent parents of the eligible person. Provided that if any person, in any or the categories at (i), (ii), or (iii) above finds place in the family card then it shall be presumed that the person is member of the Family and no further confirmation is required.*^16^

The conditionality for the appearance of one’s name in the family ration card as the sole determinant to enroll and utilize the CMCHIS excluded men and women who did not possess this document for various reasons (having no stable address, born out of unregistered marriages, newly married women whose names are yet to be included, etc.). The following case illustrates the lack of awareness among women and also norms of patrilocality^17^ that exclude women’s enrollment, since it is dependent solely on one document.

*Alamelu lived in an urban slum. She was married two years ago and wished to undergo a surgery in a private hospital using the CMCHIS. However, her name was not included in her marital home as the government was converting all paper ration cards to smart (digital) cards. She could neither use her maternal home’s ration card as her name was struck off from it as soon as she got married as per the government rules. Although one of her marital family members (a transwoman) took initiative to enroll the household in the CMCHIS, she became ineligible to utilize the CMCHIS.*

*(Alamelu, Urban, SC)*

Many other studies on PFHIS have also pointed out how the contracting out of information, education, communications (IEC) activities and enrollment to private parties have resulted in exclusions [43, 44]. The CMCHIS evaluation report also reports the lack of verifiability of the insurer’s claims of awareness activities undertaken in rural and remote areas and gaps in distribution of cards [21].

Thus, the focus on profit maximization as against social protection of the vulnerable resulted in different forms of gender specific (as in exclusion of SRH), gender intensified (poor IEC, lack of confidence to negotiate, OOPE), and gender -imposed (normative language of insurance policy) barriers.

### Gender barriers mediated by state

The State is the ultimate authority governing the CMCHIS and all its public and private partners in the healthcare and insurance industry. This section reports the barriers as identified through participant experiences that come under the control of the State in either design or execution of the program. These include the behavior of a range of street-level bureaucrats, such as enrollment officers, VAOs, ration card officers, insurance desk officers, and public and private providers who translate the policy in action. We also include in this section responsibilities of the State, such as ensuring equitable tertiary care services, gender-sensitive hospital admission norms, effective regulation of private stakeholder behavior, and governance of the scheme, the failure of which lead to an overall lack of trust among intended beneficiaries on the scheme and its parties. Many of the user experiences pointing to these gaps were cross validated in stakeholder interviews.

Narratives of women highlighted gender-imposed and intensified barriers that were sustained through State action and inaction. For women, a specific challenge that was found in the study was arranging another female attendant during hospitalization. While this is usually seen as a household barrier, this gender bias was reflected in the rules in hospitals regarding arrangement of attendants during hospitalization, which some women quoted as the reason for them delaying their surgeries.

*Palaniammal, was identified as eligible for a hip surgery during a CMCHIS health camp. Though she was enrolled in the scheme, she could not get hospitalized as the private hospital insisted on a female attendant for her admission.*

*(Palaniammal, Rural, SC)*

A similar finding on the lack of availability of adult substitutes for women within the household to seek healthcare was found in a research in Karnataka, India [45]. Neither the burden of organizing substitutes at home nor the gender of the attendants emerged as a significant barrier from narratives of hospitalized men. Holding a CMCHIS card was not helpful as it did not provide for covering the costs of arranging attendants or childcare or elderly care.

Tertiary-level hospitals, both public and private, that performed high-end surgeries and treatments, including the CMCHIS empaneled ones were, in general, concentrated in cities and towns, causing considerable travel for rural men and women. Even though district-level public hospitals that serve the rural areas were empaneled under the CMCHIS, there was shortage of health professionals or equipment, and, thus, patients were left with no choice but to go to the private hospitals.

*For accident cases, we can only do some first aid, assess the seriousness of the case, and if critical we refer them outside …. There is no private hospital also in the stretch.... It will take at least 45 min to one hour, that can cause delay and that is crucial. People here already know about it we don’t have surgeons here ... no anesthesiologist. So they won’t come here, they will go directly to private, even if they come we also will tell that only*.

*(Stakeholder Interview, Rural, Public Hospital)*

The absence of provisioning of comprehensive services at district hospitals, especially emergency services by the State, especially affected rural men who met with accidents and other emergencies.

There were many areas of dissatisfaction reported by men and women respondents, which points to the failure of the State to effectively monitor the private-party behavior under the scheme. Respondents perceived the scheme to be closely linked to political campaigns and were skeptical of their sustainability if there was a change in the ruling party. Most respondents reported an overwhelming sense of mistrust on the scheme, hospitals, and healthcare providers. Delays in admission and pre-authorization to get covered under the CMCHIS and having to produce different documents were reported by men and women.

*Will they ever see [treat] quickly? Money has not come. Money has not come in your number. How many people are waiting, you know like that in that hospital?! They keep asking the other bed occupants: “Has it come for you? Has it? Mine has not yet come [in a tone of anxiety].”*

*(Mangalamma, Rural, Other Backward Caste)*

Since women preferred to return back to their daily activities as quickly as possible and lacked financial resources, factors like perceived delays and OOPEs with the scheme card influenced decisions to avail the scheme or not. There was also a strong perception among respondents that the CMCHIS was unsuitable for emergency or critical cases (which was often the case with women seeking delayed care) compared to elective surgeries.

*Researcher: Why was the scheme not useful for you?*

*Respondent: They said it will take 20–25 days, 25 days means, how can we do operation? We can’t [wait] that much time; when needed in emergency, it is not useful it gets delayed ... Lots of signatures are needed ... from the VAO.... We have to run around for that.... If there is any big danger, even then it will take time …. If we decide to take the card and do, it* will *be late.... Now, if we have the time like one or two months, we are going to remove the uterus or something operation like that it is ok …. We cannot use the card for urgent medical needs.*

*(Vasantha, Rural, Other Backward Caste)*

These challenges made it even more difficult for women to navigate the complex processes involving the CMCHIS to access healthcare treatment. Overall, these factors lead to a mistrust of the scheme and some respondents said that they preferred to somehow mobilize money and pay upfront so that they receive good care.

The following excerpt from the interview with a stakeholder attest to this:

*People feel why take risks? Better to pay money and get good care …. Maybe they think they may not get proper treatment if they used the card.*

*(Stakeholder interview, Rural)*

Although the implementing agency of the scheme empaneled hospitals which met some basic quality criteria, there was no standardization of treatment protocols or recognition of rights of patients in the CMCHIS policy. When scheme administrators were asked about the grievances raised, they questioned the validity of these concerns as there were not many formal complaints lodged using the toll-free helpline.^18^ However, it was found that almost all interviewees were unaware of the toll-free number and their right to use it to lodge their complaints. The first author was also denied access to the grievance cell records maintained under the scheme. Respondents of in-depth interviews reported a lack of trust in the fraud-control mechanisms instituted under the scheme:

*There is no use of these [vigilance squad operating under CMCHIS]. You know the hospital staff tell us in advance not to open our mouth to anyone if they ask if we were asked to pay any money [in spite of using the CMCHIS card]*.

*(Mangalamma, Rural, Other Backward Caste)*

A healthcare activist pointed out that though monthly review meetings were held under the CMCHIS scheme, only the insurers, TPAs, and hospitals meet with government officials, whereas representatives of patient groups or civil society are not allowed to participate. He reported that there was increasing privatization and corporatization of health in the State by business giants and a stiff resistance came from medical fraternity to the TN Clinical Establishment Act & Rules. That an external evaluation of the scheme had not taken place in spite of its implementation for more than ten years needs to be also noted.

Only a few narratives indicate that the CMCHIS scheme helped women overcome financial barriers to access inpatient care. For example, one woman had to pay out of pocket even though she used her card, but she felt that the CMCHIS helped them to cut down the expenditure and “save” some money. She was unaware that she was entitled to totally cashless treatment.

*I think they took (from the card) INR 20,000 I think. Totally, it cost INR 60,000 …. Even then I have not paid INR 20,000, government paid for it …. It is a saving only … it [the scheme] is useful.*

*(Jayanthi, Urban, Other Backward Caste)*

Women who usually sought public hospitals for inpatient care did not perceive much of a benefit from the CMCHIS as they knew that they would not have to pay much from out of pocket for direct medical expenditure. Some women reported satisfaction that insurance wards in public hospitals provided better quality of care (clean surroundings, attention from providers) than general wards. It has already been discussed how the push to impose insurance to poor patients coming to public hospitals was leading to discrimination of the insured poor and uninsured poor.

## Discussion

This paper set out to fill the knowledge gaps on why fewer women than men in low-income households were utilizing the CMCHIS, a publicly funded health insurance aimed to achieve the goals of UHC. Based on the analytical framework as represented in Fig. 1, it specifically aimed to identify gender-based barriers that play out across households, communities, markets, and State in each stage of the scheme cycle (from design, implementation to impact). It also aimed to understand the role of the CMCHIS as a UHC scheme to remove financial barriers faced by the poor to access healthcare.

Based on the findings of a study presented in this paper, we argue that the CMCHIS has allowed continuance of gender barriers of financial as well as non-financial nature in all of the societal institutions—household, community, market, and State. If the intent of public policies is to improve access to health for the poor and reduce financial burden this policy has fallen short on both counts. The findings on poor financial protection resonate with previous studies on the limited impact of PFHIS [46–48], including in TN [22, 49] and a few which have looked at utilization specifically by women [50, 51]. By highlighting gender as a social determinant of health and how gender barriers sustained by a health policy permeate health systems, this paper provides one of the first and comprehensive insights on processes and practices that mediate access, utilization, and impact of UHC schemes on gender and health equity.

The household is not just a site of inequality but also plays a role in shaping gender relations by laying down rules, assigning activities, providing control over resources often making health access pathways for women different from that of men. As the literature review [20] revealed, research and evaluation of most PFHIS so far have considered the entire household as an egalitarian unit without recognizing the unequal intra household dynamics. This paper explored the intra-household distributional inequities that create several barriers for women, such as mobility constraints, need for permissions, double burden (continuous and invisible) of unpaid care work, financial dependence, and rationing of care. While women’s illnesses receive lesser priority than men’s illnesses in general among poor households, men too delay their hospitalizations for fear of losing wages and they also choose public hospitals over private to reduce health expenditures. Women in some better-off households choose a private hospital for perceived better quality and quicker delivery of services, even if they had to spend more. Age plays an important intersectional role as biologically, women outlive men but are socially and financially dependent on other family members. An elderly male respondent in the study was able to mobilize resources for his treatment by pawning his recently wed daughter-in-law’s jewels, whereas an elderly widow living with two sons chose a faraway public facility over a private facility for fear of burdening household resources.

Women not only have to subscribe to norms within their households but also as laid down by the spatial, class-based, caste-based, and political structures in the community. The paper demonstrates that for poor and marginalized women, access to information and benefits of social protection schemes are limited depending on how well they are able to surmount household- and community-level barriers. In relying solely on ration cards for determining eligibility, the findings highlight how the scheme overlooks the patrilocal and patriarchal nature of Indian societies, where women shift from natal to conjugal families but their documentary proofs (like ration cards) do not get transferred easily. The enrollment process clearly excludes marginalized women who do not live in family arrangements as defined by the insurance companies.

The CMCHIS enrollment activities were found to create exclusions and sustain the vulnerabilities of those who needed social protection the most. Thakur [52] in the Karnataka (India) study on health insurance schemes also observed that social patterning in the failings of IEC in PFHIS, which led to exclusions of vulnerable groups.

Even when women manage to surpass the household and community barriers to seek inpatient care, they confront inadequate availability of health services on the one hand and a complex maze of hospital and insurance provider procedures to be navigated on the other. It has to be noted that the CMCHIS is mounted in a PPP mode, where the private sector is unregulated and inequitable [23, 53, 54].

The CMCHIS is based on the principle of “strategic purchasing”, whereby the government, instead of directly providing the services, purchases the service from private or public entities through an insurance underwriter. These arrangements involve multiple stakeholders who bring their own specific interests and control certain aspects of the policy implementation [55]. Street-level bureaucrats, such as the VAOs, ration officers, district kiosk members, hospital representatives, and healthcare providers, create further barriers and delays by using their discretionary powers to suit their stakes in the policy [56]. The paper highlights how women have to abide by gender-based norms on hospital admissions, produce documentary proof, and still face denials in coverage in hospitals because of the narrow insurance packages with a focus on high-end tertiary care. With poor monitoring by the State of public and private hospitals, combined with low awareness among women on their entitlements under the scheme, women continue to either be excluded or may be inappropriately included in treatment plans. With very little changes to OOPEs even while using the CMCHIS, the paper documents women’s lack of trust in, acceptance of, and consequent poor utilization of the scheme. The processes cumulatively push women towards unempaneled private providers, which causes financial stress.

The State has subscribed to market principles that dictate what and whom the publicly funded health insurance policy includes (or excludes). To be a gender-equitable policy, the CMCHIS is expected to cater to both similar (horizontal) and different (vertical) health needs and reach the furthest first (women, especially marginalized). However, the scheme did not adequately cover SRH or chronic diseases with recurring expenditures that caused considerable financial burden. Women became victims of treatment decisions based on information asymmetry^19^ that became emboldened with insurance processes. Private providers resort to “cherry picking”^20^ of cases under the CMCHIS that help meet their targets and would benefit them.

A more disturbing finding concerns how a market based public–private arrangement was weakening the existing social protection available through public health systems. Most of the poorest economic quintiles, SCs, and women prefer to seek healthcare in the public health institutions in TN [9, 25] for all major ailments, despite limitations. With the advent of the insurance system, market ideologies of “choice” and “competition” determine the provision or denial of care for women in public and private health sector. This is linked to women’s insurance status and type of treatment required, which would or would not generate revenue for the hospitals. It needs to be remembered that hysterectomies were found to be overutilized in the earlier version of TN insurance scheme [26] and were later made as a reserved procedure available only in public hospitals through the CMCHIS. The recent evaluation report indicates that provider-induced treatments could be taking place even inside public hospitals under the CMCHIS to meet targets [21]. Further research is needed to explore how treatment decisions of hysterectomy, caesarian, appendectomy, and such procedures known for provider-induced utilization are made in public hospitals for insured patients. These are serious gender concerns as the study showed that women and poor are more likely to choose public facilities.

Apart from unraveling gender-based inequities, this paper has traced healthcare access pathways which are dynamically shaped at the intersections of geography, class, caste, age, marital status, disability status, and sexual orientation. Intersectionality, which has its roots in Black feminism and critical social theory [57], can illuminate the interactions of different social positions in understanding of human experience [7].

We already saw how some barriers were common for both men and women but were more acutely experienced by women. These include delayed hospitalizations, poor awareness, stigma associated to caste group, etc. The health program and systems actively contributed to the intersecting axes of privilege and oppression. For instance, while women are more likely to be at disadvantage for not having domiciliary proof (ration card) simply because of the cultural practice of women changing their homes and identities, migrant and homeless men and sons born to women outside “legal” marriages were also likely to be excluded from ration cards and thereby from the CMCHIS. Thus, gender blindness in policy allowed a gender-specific barrier become an institutionalized, imposed barrier.

The paper demonstrates that the conceptual framework based on SR ideas helped to deconstruct the social determinants of health equity, capture their interrelations, and link them to structural factors in the context of an individual seeking healthcare through PFHIS. Specifically, the nuances of a market-based system distorting the State from its welfare objective and its implications for gender and health equity was unearthed using this framework. Though the authors did not decide on using the intersectionality lens apriori, the findings have reiterated the importance of shedding the use of a solitary lens, of treating human experiences as shaped by only one or two or composite social positions. We strongly advocate for the integration of intersectionality as an analytical category while unpacking the institutional elements in the conceptual framework and as a methodological category for future research. It might also be useful to factor in the role of the street-level bureaucrats who, according to Lipsky [41] p13, “effectively become the public policies they carry out”. Though Lipsky’s framework restricted to public service workers, in PPP models like the CMCHIS, we need to include private sector representatives such as hospital receptionists, administrators, doctors, nurses, and liaison officers. The theories of street bureaucracy can help unearth why and how discretionary powers to accept/reject and include/exclude claim entitlements are applied and what strategies are needed to minimize the asymmetry of power and plug the gaps created in translating the policy goals into outcomes.

Overall, the paper throws light on gender biases operating in overt and covert ways in the norms of the CMCHIS design and implementation. Households, along with the institutions of community, State, and market, shape intersecting SR to sustain gender barriers that affect pathways to financial protection in the CMCHIS. These aspects covered in this paper explain the low utilization of the scheme particularly by women.

### Policy and research recommendations

For social policy to be transformative, it needs to relate to power imbalances in society that encourage, create, and sustain vulnerabilities [58]. PFHIS can become transformative if it supports the individual eligibility of women as citizens to directly participate in the scheme [59, 60]. The scheme needs to delink its access from the living arrangements or marital relationships of individuals. For example, the scheme should not make it difficult for a woman who lives with a parent or is not legally married but lives with partner, or for a transgender who lives with a partner to enroll or utilize the scheme. Each enrolled individual could be provided with one entitlement card rather than one per household. Alternate types of documentary evidence have to be allowed instead of relying only on ration card.

An ongoing IEC strategy that uses multiple channels (mass and interpersonal) of communication needs to be devised to ensure that unbiased and reliable information reaches everyone regardless of education, income, language, or location. Awareness and enrollment activities to be planned to keep in mind time, money, and mobility constraints, especially of women. The processes of enrollment have to be quick and transparent. The onus of verifying a person’s entitlement has to be with the health system and not with the individual needing healthcare after they reach the healthcare facility.

In terms of the scheme package, there needs to be an explicit inclusion of a range of SRH services as in the Thailand UHC scheme [61], outpatient consultations, and equal emphasis on preventive measures. Insurance packages need to cover costs of childcare or elderly care, provision for attendants during hospitalization, loss of wages for both men and women, and transportation.

Accountability mechanisms have to be reinstated in the scheme to check unnecessary charges and inappropriate service provisions. All healthcare decisions under PFHIS have to be informed, understood, and voluntary. Private health systems need to be regulated effectively so that marginalized members are not exploited for commercial interests. Laying down standard treatment protocols, price regulation, and stringent punishments would be necessary to avoid ethical violations. The State has to improve its governance of the scheme, improve accountability of stakeholders, and constitute committees that involve civil society groups and gender experts. An effective grievance redressal mechanism need to be reinstated.

The spirit of the UHC scheme has to be to confer right to healthcare fairly and justly rather than allowing “money to follow the patient”. Government has to reorient itself to strengthen public spending and public health systems to reach those with priority needs and facing multiple levels of intersecting inequities [19]. A legally enacted right to health can go a long way in translating the entitlements to tangible actions.

At the research level, this paper has revealed that much of the complexities of a PFHIS at the design, implementation, and utilization levels still remain hidden. There is a need for building evidence base through health system policy research on the how UHC policies impact different marginalized individuals and groups. Research could focus on case studies that touch upon all the six building blocks of health systems suggested by World Health Organization (WHO) (2004) [62] of a specific UHC plan. Such research needs to necessarily employ a rights-based social justice approach. In terms of research methods, participatory methods and qualitative methods, such as ethnography and phenomenology, can be employed to complement econometric and quantitative methods. There is a need to explore the intersectional experiences within and outside the household, including those of socially excluded groups like the differently abled, sex workers, lesbian/gay/bisexual/queer/intersex, institutionalized members, adolescents, homeless, migrants, and so on. It is also important to recognize that low-income lower-caste men, possibly those of religious minorities (that are, Christians and Muslims) also face significant challenges in accessing care. Therefore, a study, through the lens of intersectionality of men’s access and uptake of services under the scheme is recommended. Research is also needed with street-level bureaucrats, senior bureaucrats, administrators, private hospitals, and insurance representatives to provide deeper insights into the challenges of implementing the scheme.

### Limitations

The paper has some limitations. The researcher faced challenges in collecting data from men as they were mostly available only in late evenings and some were found to be intoxicated, which limited the female researcher to 16 interviews. Also, findings from this paper may not be generalizable to locations that are not similar to the study sites selected for this research. A larger research is needed to validate some of the findings found in this paper.

## Conclusions

The interplay of gender within publicly funded health insurance programs has not been adequately explored in research, especially in LMICs. So far, the lack of gender lens in health policy analyses has rendered the experiences of women from low-income and marginal categories accessing healthcare invisible, which the paper has attempted to bring to light. Using the case of TN’s CMCHIS (India), this paper has explored the implications of gender power relations operating and interacting in the different institutions of household, community, market, and State spheres in mediating barriers on access pathways to healthcare. The paper shows that the support women received from the state health insurance scheme have remained gender iniquitous. It did not eliminate barriers within the households and communities, but reinforced barriers through its gender-blind market orientation and implementation.

In terms of methodological and theoretical contributions, this paper has demonstrated the use of Naila Kabeer’s gender analytical framework, which argues to look at gender not as a standalone category but as a relational concept, which permeates different institutions. We have engaged with feminist critique of the scheme emerging from socialist feminism that questions unitary household model, argues for recognizing women’s citizenship rights, emphasizes the need for policies to recognize “sameness” and “difference” among people, acknowledge the standpoint theory in reflecting the voices of women on the insurance scheme, and lastly, incorporate the concept of “intersectionality” to understand how multiple subject positions interact to produce different experiences.

PFHISs, which are gender blind when implemented within an inequitable and unaccountable health system, disempower the poor and worsen the condition of poor and marginalized women. UHC schemes set within national health policies, which address social determinants of health with a human rights framework and gender sensitivity, are a necessity to achieve health equity.

## Supplementary Information

**Additional file 1: Table 4.** Codes, Subcodes, Categories, Themes. Summary of Codes, Subcodes, Categories, and Themes from Content Analysis

**Additional file 2.** Profile of In-Depth Interview Male Respondents. Age, Religion, Caste, Marital Status, Current Occupation, Reason for Hospitalization, Type of Facility for Hospitalization

**Additional file 3.** Profile of In-Depth Interview Female Respondents. Age, Religion, Caste, Marital Status, Current Occupation, Reason for Hospitalization, Type of Facility for Hospitalization

**Additional file 4: Table 6.** Institutional Analysis of the CMCHIS. Institutional analysis of the CMCHIS using the SR Framework listing the components of five dimensions (rules, activities, resources, people, power) of each of the four institutions (household, community, market, State), gender-based barriers and outcome on access to healthcare under the CMCHIS.

**Additional file 5.** In-Depth Interview Guidelines for men and women. Guidelines used by first author during in-depth interviews with men and women.

## Abbreviations

CMCHIS: Chief Minister’s Comprehensive Health Insurance Scheme
IEC: Information, Education, Communications
INR: Indian Rupee
IRB: Institutional Review Board
LMIC: Lower-Middle Income Countries
OOPE: Out-of-Pocket Expenditure
PFHIS: Publicly Funded Health Insurance Scheme
PPP: Public–Private Partnership
SC: Scheduled Caste
SR: Social Relations
SRH: Sexual and Reproductive Health
TN: Tamil Nadu
TPA: Third Party Administrators
UHC: Universal Health Coverage
VAO: Village Administrative Officer
WHO: World Health Organization

## Footnotes

1 In September 2018, all but a few states integrated their schemes with the Ayushman Bharat Pradhan Mantri - Jan Arogya Yojna (AB PM-JAY), which is funded by the Government of India.

2 The larger doctoral study also included secondary data analysis, a quantitative primary research, discussing the results of which are beyond the scope of this paper

3 For further description of SR framework, see [28, 29].

4 As of 2018.

5 The Tamil Nadu Clinical Establishments (Regulation) Act of 1997 was amended and Tamil Nadu Clinical Establishments (Regulations) Rules, 2018, were published in June 2018.

6 As per the CMCHIS website (wwww.cmchistn.com) accessed on 15 May 2015.

7 Ration card is given in the name of the head of the household, usually male, with information of all eligible members who are co-residing under the same roof and share a common hearth and purse. See http://www.tncsc.tn.gov.in/html/pds.htm.

8 Government of India classifies socially disadvantaged groups as Scheduled Caste, Scheduled Tribes, and Other Backward Castes (including Muslims). Schedule Caste refers to a social group at the bottom of the hierarchy, historically marginalized and given special status as part of affirmative action as per the Constitution of India.

9 http://www.equator-network.org/reporting-guidelines/srqr/.

10 All names in this paper are fictitious.

11 https://stats.oecd.org/Index.aspx?datasetcode=TIME_USE.

12 These refer to women married in paperless second marriages, which, during the subsistence of the first marriage, is illegal in India. Second wives do not get any protection under law.

13 Kiosks were set up by State in all district collector offices for round the year enrollment of eligible households in the CMCHIS even if they missed an enrollment camp.

14 In India, Hinduism is the majority religion followed by Islam, Christianity, and others.

15 Houses with thatched roofs and walls.

16 https://www.cmchistn.com/eligibility_en.php.

17 The cultural practice whereby woman moves and starts living with her husband’s family upon marriage.

18 https://www.vikatan.com/oddities/miscellaneous/144606-rti-about-ayushman-bharat.

19 Information asymmetry in health refers to healthcare providers possessing more information about illness than the patient. For more information, see https://web.stanford.edu/~jay/health_class/Readings/Lecture01/arrow.pdf.

20 Selectively focusing on patients easy to reach or whose cost of care can help benefit the provider or health facility.
