# Supplementary material for: Why is women’s utilization of a publicly funded health insurance low?: a qualitative study in Tamil Nadu, India
Source: BMC Public Health. 2021 Feb 12;21:350. doi: 10.1186/s12889-021-10352-4 (PMC7881649; doi:10.1186/s12889-021-10352-4)
Supplement: Supplementary file 1 — Additional file 1: Table 4. Codes, Subcodes, Categories, Themes. Summary of Codes, Subcodes, Categories, and Themes from Content Analysis [file 12889_2021_10352_MOESM1_ESM.docx]

| **Table 4 Codes, Subcodes, Categories, Themes** | | | | | | | | |  |
| --- | --- | --- | --- | --- | --- | --- | --- | --- | --- |
|  | ***Household*** | | ***Community*** | | ***Market (Health)*** | ***Market (Insurance)*** | ***Market*** | ***State (Policy)*** | |
|  | ***Subcodes*** | ***Codes*** | ***Subcodes*** | ***Codes*** | ***Subcodes*** | ***Subcodes*** | ***Codes*** | ***Subcodes*** | ***Codes*** |
| ***Activity*** | Delivery; child care; elderly care; sick care; domestic work; paid work; domicile change | Continuous care work; invisible | Women confined to home; men form groups; camps | Less awareness of outside world | Profit motives; high-end care; treatment as per packages; urban pockets | Packaged healthcare; cover high-cost, low-frequency illness; awareness camps, enrollment; card distribution | Excluding the less profitable but needed activities | Strategic purchasing; access; financial protection; social protection; public health | Changing from provider to purchaser; contracting out as panacea for poor performance of public sector |
| ***People*** | Male; female; spouse; wife; widow(er); unmarried; single; deserted; elderly; dependents; children | Intra-household differences; multiple positions | Scheduled caste; other backward caste; colonies; female-headed households; panchayat; political party members; poor; not poor | Networks with authority | Businesses; corporates; specialists; doctors; nurses; administrators | Commercial insurers; TPAs; vendors | Outsourcing leading to multiple players translating policy | Politicians; street bureaucrats; revenue officials; kiosk officers; civil society; doctors; nurses; liaison officers; other healthcare providers | Health as a political and commercial subject |
| **Resources** | Liquid cash; savings; cattle; land; jewels; loans; asset selling; maternal support; insurance; employers | Unequal distribution; distress coping | Information; land; welfare schemes; temple entry; shared toilet | Competition for benefits | Pricing of services; over utilization; subsidies; margins from the CMCHIS packages; out-of-pocket expenditures | Premium; one card per family; card distribution; marketing in easy areas; administrative costs | Profits based on performance targets | Health budgets; international funding; tertiary hospitals; secondary hospitals; primary facilities; tax money; equipment; vacancies; | Tax money routed to private health and commercial insurance |
| ***Rules*** | Unequal healthcare; substitute for domestic work; sacrifice; ration; attention to males | Women put others over self | Maintain hierarchy; informal network; favors; influence | Excluding the lowest in hierarchy | Covered illness; profitable cases; cash advance; charge above the CMCHIS; deny low packages | Increase revenue; exclude covering drugs, care work, and SRH; competition; pre-authorization delays; exclude “illegal” family members | Illusion of choice but exploitation of customers | Promote PPP; citizenship; ration card; patrilocality; hospital admission rule; poor grievance redressal | Households as target units; citizens as passive recipients of healthcare; lack of rights-based approach |
| ***Power*** | Health decisions; negotiate; bargain; discriminate; domestic violence | Lack of autonomy among vulnerable in decision-making | Discriminate; sanction; “outsiders”; “safe/unsafe” | Perpetuation of stigma | Compelling enrollment; deny care; selective information | Distort provider behavior; resist regulation by State | Poor accountability | Overall controlling authority; policy making; funding; monitoring; evaluation; political will; trust | Poor governance; lack of trust among citizens |
| **Subcategory: Type of Gender-Based Barriers** | Gender Specific | | Gender Specific and Gender Intensified | | Gender Specific; Gender Intensified; Gender Imposed | | | Gender Specific; Gender Intensified; Gender Imposed | |
| **Subcategory: Stage in Scheme Cycle** | Design; awareness; enrollment; utilization; impact | | Design; awareness; enrollment | | Design; awareness; enrollment; utilization | | | Design; awareness; enrollment; utilization; impact | |
| **Themes** | **Double burden of care work; rationing of healthcare** | | **Exclusions of most marginalized** | | **Increase inequities; exploitation of marginalized; profits over protection; gender blindness** | | | **Complex pathways; weakening of public health; gender blindness** | |
