# Supplementary material for: Why is women’s utilization of a publicly funded health insurance low?: a qualitative study in Tamil Nadu, India
Source: BMC Public Health. 2021 Feb 12;21:350. doi: 10.1186/s12889-021-10352-4 (PMC7881649; doi:10.1186/s12889-021-10352-4)
Supplement: Supplementary file 2 — Additional file 2. Profile of In-Depth Interview Male Respondents. Age, Religion, Caste, Marital Status, Current Occupation, Reason for Hospitalization, Type of Facility for Hospitalization [file 12889_2021_10352_MOESM2_ESM.docx]

| **Profile of Male In-Depth Interview Respondents (N=16)** | |
| --- | --- |
|  |  |
| ***Age (in years)*** |  |
| Mean age | 54 |
| Youngest | 35 |
| Oldest | 80 |
| ***Caste*** |  |
| Scheduled Caste | 7 |
| Other Backward Caste | 9 |
| ***Religion*** |  |
| Hindu | 12 |
| Muslim | 2 |
| Christian | 2 |
| ***Current Occupation of Men*** |  |
| Not employed anywhere | 5 |
| Self-employed (potter, street vendor) | 4 |
| Regular wages (work in shops) | 1 |
| Casual labor (non-agricultural) | 5 |
| Casual labor (agricultural) | 1 |
| Stopped due to illness | 5 |
| ***Reason for Hospitalization*** |  |
| Cardiovascular diseases | 7 |
| Fever | 2 |
| Pain in hips/legs (neuro/muscular/skeletal) | 5 |
| Accident | 1 |
| Gastrointestinal | 1 |
| Total respondents with chronic ailments | 12 |
| **Type of Facility** |  |
| Public hospital | 7 |
| Private hospital | 9 |
