# Supplementary material for: Why is women’s utilization of a publicly funded health insurance low?: a qualitative study in Tamil Nadu, India
Source: BMC Public Health. 2021 Feb 12;21:350. doi: 10.1186/s12889-021-10352-4 (PMC7881649; doi:10.1186/s12889-021-10352-4)
Supplement: Supplementary file 3 — Additional file 3. Profile of In-Depth Interview Female Respondents. Age, Religion, Caste, Marital Status, Current Occupation, Reason for Hospitalization, Type of Facility for Hospitalization [file 12889_2021_10352_MOESM3_ESM.docx]

| \| **Profile of In-Depth Interview Female Respondents (N=33)** \| \| \| --- \| --- \| \|  \|  \| \| ***Age (in years)*** \|  \| \| Mean age \| 44 \| \| Youngest \| 22 \| \| Oldest \| 70 \| \| ***Caste*** \|  \| \| Scheduled Caste \| 11 \| \| Other Backward Caste \| 22 \| \| ***Religion*** \|  \| \| Hindu \| 26 \| \| Muslims \| 5 \| \| Christians \| 2 \| \| ***Marital Status*** \|  \| \| Married/living with spouse \| 20 \| \| Widow \| 8 \| \| Separated/unregistered marriage \| 3 \| \| Unmarried \| 2 \| \| ***Current Occupation*** \|  \| \| Homemaker \| 19 \| \| Self-employed (potter/tailor/caterer) \| 5 \| \| Regular wages \| 1 \| \| Casual labor (non-agricultural) \| 5 \| \| Casual labor (agricultural) \| 2 \| \| Stopped due to illness \| 5 \| \| ***Reason for Hospitalization*** \|  \| \| Cardiovascular diseases \| 6 \| \| Fever (unidentified, typhoid, cholera) \| 4 \| \| Pain in hips/legs (neuro/musculoskeletal) \| 5 \| \| Thyroid disorder/lung disease/skin disease/varicose veins \| 4 \| \| Breast cysts: malignant and benign \| 3 \| \| Uterine cysts: malignant and benign \| 3 \| \| Kidney stone \| 2 \| \| Stomach ulcers \| 2 \| \| Accident/fracture/gallbladder stone/hernia \| 2 \| \| Total respondents with chronic ailments \| 21 \| \| ***Type of Facility*** \|  \| \| Public hospital \| 17 \| \| Private hospital \| 16 \| | |
| --- | --- | --- | --- | --- | --- | --- | --- | --- | --- | --- | --- | --- | --- | --- | --- | --- | --- | --- | --- | --- | --- | --- | --- | --- | --- | --- | --- | --- | --- | --- | --- | --- | --- | --- | --- | --- | --- | --- | --- | --- | --- | --- | --- | --- | --- | --- | --- | --- | --- | --- | --- | --- | --- | --- | --- | --- | --- | --- | --- | --- | --- | --- | --- | --- | --- | --- | --- | --- | --- | --- | --- | --- | --- | --- | --- | --- | --- | --- | --- |
|  |  |
|  |  |
|  |  |
