# Supplementary material for: Why is women’s utilization of a publicly funded health insurance low?: a qualitative study in Tamil Nadu, India
Source: BMC Public Health. 2021 Feb 12;21:350. doi: 10.1186/s12889-021-10352-4 (PMC7881649; doi:10.1186/s12889-021-10352-4)
Supplement: Supplementary file 4 — Additional file 4: Table 6. Institutional Analysis of the CMCHIS. Institutional analysis of the CMCHIS using the SR Framework listing the components of five dimensions (rules, activities, resources, people, power) of each of the four institutions (household, community, market, State), gender-based barriers and outcome on access to healthcare under the CMCHIS. [file 12889_2021_10352_MOESM4_ESM.docx]

|  | **Table 6 Institutional Analyses of the CMCHIS** | | | | | |  |
| --- | --- | --- | --- | --- | --- | --- | --- |
| **Institution** | **Rules** | **Activities** | **People** | **Power** | **Resources** | **Outcome on access to healthcare for women under the CMCHIS** | **Type of gender barriers** |
| **1)Household** | Men are head of households (even if not living with family) and women are subordinate to men. | Men engage in paid work outside home while women engage in reproductive and unpaid care work (even while engaged in paid work). | Usually consists of a married couple living with children. The elderly, sick, unmarried, and separated live as dependents | Decision-making power rests with men in all matters of household except in women-headed households. | Men are entitled to and control material and non-material resources while women need to negotiate and bargain. | Women need to seek permission to go out; arrange escorts, substitutes, and attendants; and face difficulties in mobilizing finances to seek treatment. Men's treatment is prioritised. Women's names are missing on ration cards and CMCHIS cards. Unmarried, single, elderly, and disabled women face more barriers. | Gender Specific |
| **2)Community** | Social groups (based on religion, class, caste, or political affiliation) within the community occupy different hierarchical positions. | Members of a social group live closer to each other, marry within the group, engage in traditional occupations, and minimally interact with non-members. Usually only men participate in activities held outside homes. | Groups are characterized by strong social identities and interaction with outsiders not encouraged. Those who belong to lower social, economic groups are stigmatized. Absence of formal civil society groups leads to poor representation of common concerns. | Panchayat leader or political members, are mostly male and from upper castes. They influence how social benefits are distributed. Households with connections to such people able to negotiate access to benefits. | There is unequal distribution of resources (land, water, toilets, temples, information, welfare measures) and competition across social groups. | There is competition between social groups to access the CMCHIS benefits, which often favor dominant groups. Women have mobility constraints due to gender and caste norms that mediate participating in scheme activities. Women in marginalized social groups who are not living in male-headed households are stigmatized and have accentuated disadvantage in access to resources. Government does not involve people in the CMCHIS. | Gender Specific and Gender Intensified |
| **3)Market** | It packages healthcare as commodities and promotes an illusion of “choice for customers”. | It charges for the health services arbitrarily. Customers are cherry picked to maintain profit margins. | Hospitals employ specialists and super specialists to provide sophisticated care, mainly in urban areas and promote business interests. Insurers subcontract to TPAs and vendors. | Acute information asymmetry between patients, doctors, and insurers, where the patient is the weakest stakeholder. Markets unregulated by the State dictate the availability, pricing of healthcare services, and influence public health systems through PPPs. | PPPs like the CMCHIS become a source of revenue for private health and insurance industries. Insurers conduct superficial awareness and enrollment; they restrict enrollment by narrowing family definitions to maintain profits. Private hospitals impose cash advances and OOPE. | By design, the CMCHIS focuses on high-end curative treatments. Insurance excludes outpatient services, high-frequency, low-margin health and services needed by women. Cost of awareness and enrollment activities had to be kept low and excluded remote areas, marginalized people and women who lack documents. Undue emphasis is placed on revenues and competition causes distortion in provision of care. | Gender Specific, Gender Intensified, and Gender Imposed |
| **4)State** | Increasing withdrawal from providing social protection to the weak and marginalized. | Instead of providing health services directly, the State increasingly gives out contracts to private parties through “strategic purchasing” with poor regulation. | Scheme administrators and frontline workers use discretion and are not sensitive to gender concerns. They are compelled by political pressures and business interests. Processes are not transparent to civil society. | The State is capable but fails to control the private health and insurance market. Provider-induced utilization is accepted. The State’s social protection commitment is weakened by market ideologies and international pressures. | The State lacks fiscal space and political will to allocate adequate funds for financing healthcare, especially public health. | Public and private health systems are inequitably distributed and unregulated, leading to exclusions and OOPEs affecting women, poor, and marginalized castes the most, even with the CMCHIS. Gender-biased norms in insurance is attested by the State in the CMCHIS’s narrow design and implementation. The CMCHIS is poorly governed, resulting in complexities and eroding trust scheme among beneficiaries. | Gender Specific, Gender Intensified, and Gender Imposed |
