# Supplementary material for: Why is women’s utilization of a publicly funded health insurance low?: a qualitative study in Tamil Nadu, India
Source: BMC Public Health. 2021 Feb 12;21:350. doi: 10.1186/s12889-021-10352-4 (PMC7881649; doi:10.1186/s12889-021-10352-4)
Supplement: Supplementary file 5 — Additional file 5. In-Depth Interview Guidelines for men and women. Guidelines used by first author during in-depth interviews with men and women. [file 12889_2021_10352_MOESM5_ESM.docx]

**Guidelines for In-Depth Interviews with Women and Men**

**Broad areas of enquiry:**

1. ***Background details/Rapport building***

- Current household composition
- Nature of paid and unpaid work, regularity of income, assets, savings, debts of household and of women in particular
- Marital and reproductive history (women)
- Major health issues, deaths, morbidities among all family members

***(B) General health-seeking patterns, specifically for women, elderly, disabled etc.***

- Places of treatment when someone is sick
- How expenses are managed when a woman falls sick and a man falls sick in the household?
- What factors are considered when someone is sick and needs healthcare?
- Choice of different healthcare providers

***(C)*** ***Events surrounding the main morbidity which is focus of study***

- When did the initial symptoms occur and were recognized?
- What kinds of places you have gone and consulted for this illness? What happened in each stage and setting?
- Discussions that happened at the household between different members at every stage to take the necessary decisions
- Kinds of direct and indirect expenditure and costs because of these healthcare visits
- How these expenditures were met
- Other adjustments and support needed to make these healthcare visits

***(D)*** ***Awareness and experiences of CMCHIS enrollment and utilization***

- Knowledge of insurance, specifically, health insurance
- Knowledge of the CMCHIS
- How was your household enrolled?
- What are the reasons for your household not being enrolled? (select cases)
- When and how was it decided that the CMCHIS card could be used for hospitalization?
- Experiences inside the hospital setting regarding the use of the card during the admission process
- Reasons why hospitalization was not covered under the CMCHIS (select cases)
- Describe the expenditures you/household had to pay during the hospitalization (direct, indirect, household)
- Experiences during hospitalization (other than medical expenditure)
- Health and health expenditures post discharge
- Grievance or complaint about the use of the CMCHIS card during the hospitalization, whether expressed

***(E) Perceptions of the CMCHIS***

- Usefulness
- Relevance to self and others According to you, how has the CMCHIS scheme helped in your journey to seek treatment and get better?
- Any change in autonomy, access, choices
- Suggestions for improving the scheme
